# The Role of Plant-Based Beverages in Nutrition: An Expert Opinion

**DOI:** 10.3390/nu17091562

**Published:** 2025-04-30

**Authors:** Joanna Rachtan-Janicka, Danuta Gajewska, Hanna Szajewska, Dariusz Włodarek, Halina Weker, Katarzyna Wolnicka, Klaudia Wiśniewska, Piotr Socha, Jadwiga Hamulka

**Affiliations:** 1 Department of Functional and Organic Food, Institute of Human Nutrition Sciences, Warsaw University of Life Sciences (SGGW-WULS), 166 Nowoursynowska Street, 02-787 Warsaw, Poland; 2 Department of Dietetics, Institute of Human Nutrition Sciences, Warsaw University of Life Sciences (SGGW-WULS), 166 Nowoursynowska Street, 02-787 Warsaw, Poland; dariusz_wlodarek@sggw.edu.pl; 3 Department of Pediatrics, The Medical University of Warsaw, 02-091 Warsaw, Poland; hszajewska@wum.edu.pl; 4 Department of Nutritional, Institute of Mother and Child, 01-211 Warsaw, Poland; halina.weker@imid.med.pl; 5 Independent Researcher, 02-736 Warsaw, Poland; katarzyna.wolnicka@wum.edu.pl; 6 Department of Social Medicine and Public Health, Medical University of Warsaw, 02-091 Warsaw, Poland; klaudia.wisniewska@wum.edu.pl; 7 Department of Gastroenterology, Hepatology, Nutritional Disorders and Pediatric, The Children’s Memorial Health Institute, 04-730 Warsaw, Poland; p.socha@ipczd.pl; 8 Department of Human Nutrition, Institute of Human Nutrition Sciences, Warsaw University of Life Sciences (SGGW-WULS), 02-787 Warsaw, Poland; jadwiga_hamulka@sggw.edu.pl

**Keywords:** plant-based beverages, nutrients, fortification, health benefits, risk, diet

## Abstract

The market of plant-based food, including plant-based beverages, is one of the fastest-growing food sectors within the food industry and a subject of major research in the area of new product development. Plant-based beverages are a diverse group of non-dairy beverages with varying nutritional value, depending on the raw material sources and additional substances used in their production. A wide range of plant beverages makes it possible to choose products tailored to individual consumer preferences and needs as a part of sustainable dietary patterns. Increased consumer awareness of the environmental and health implications of proper nutrition, interest in plant-based diets, climate, and natural resource protection, as well as ethical concerns about animal welfare and the negative environmental impact of animal production, have led some consumers to seek a more balanced diet based on varied plant-based products, including beverages. Considering the highly diversified nutritional value of plant-based beverages, their availability, convenience, accessibility to consumers, ethical and environmental concerns, increasing health concerns as well as growing popularity of plant-based beverages as potential cow-milk alternatives, the Group of Experts in medicine and nutritional sciences presents the opinion on the nutritional value, health benefits and concerns of the available plant-based beverages. The opinion was based on a critical review of the current scientific literature, as well as on the experts’ experience. This knowledge can be used to make the right choices to improve the nutritional status and health of the consumers from different groups. Since the nutritional profiles of plant-based beverages vary across different plant-based drink varieties and they do not have standards of identity, in our opinion, there is a need for action to standardize nutrient fortification regarding the type and amount of added ingredients to ensure the safety of consumers and avoid potential over- or under-fortification of plant-based beverages.

## 1. Introduction

The nutraceutical/functional food market is one of the fastest-growing segments among foods with potential health-promoting effects. Plant-based beverages hold a special place in this market and have now attracted the interest of food producers (the food industry), the scientific community, and a wide range of consumers [1,2]. Increased consumer awareness of the environmental and health implications of proper nutrition, interest in plant-based diets, climate, and natural resource protection, as well as ethical concerns about animal welfare and the negative environmental impact of animal production, have led some consumers to seek a more balanced diet based on more plant-based products (e.g., vegetarian, vegan, and flexitarian) [1,2,3,4]. Plant-based beverages are also naturally lactose-free, making them suitable for people with lactose intolerance. Fortified drinks contain additional vitamins and minerals, such as calcium, vitamin D, and vitamin B_12_, which are important for bone and overall health [1,2,3,4]. These factors have resulted in a dynamic growth of the global market for plant-based beverages, estimated at 15% per year between 2023 and 2028 [5]. New beverages based on plant raw materials are constantly appearing, with a variety of flavors, fermented, and fortified with calcium, vitamins D, B_12_, and B_2_, prebiotics, and instant products.

On the one hand, the market for these products is rapidly growing, their popularity is increasing, and many consumers are choosing to partially or fully replace cow’s milk with plant-based beverages. On the other hand, it is unknown how this will affect society’s health in the long term. In view of these trends and uncertainties, the Expert Group presents an opinion on the nutritional and functional properties of the available plant-based beverages compared with cow’s milk based on current scientific evidence.

This critical review was primarily designed to analyze the nutritional value of milk and several plant-based beverages with a focus on macro- and micronutrient content. The second objective was to analyze the health benefits and risks of consuming the different beverages, and on this basis to formulate recommendations for different groups of consumers. This will allow for a better definition of the place of plant-based beverages in a well-balanced diet in different population groups.

### 1.1. Definition and Types of Plant-Based Beverages

#### 1.1.1. Definition

Plant-based beverages, also referred to as dairy-free or alternative beverages, are a group of drinks prepared from raw plant materials such as cereal grains and pseudo-cereals (e.g., buckwheat or quinoa), seeds, nuts, legumes, and potatoes. Because they resemble cow’s milk in appearance, consistency, and density, they are quite often incorrectly referred to as plant-based milk or vegan milk [4,6,7].

Plant-based beverages are liquids that are produced by aqueous extraction processes of plant material through decomposition (reduction of the particle size of the raw material) and/or homogenization. The resulting particles range from 5 to 20 μm in size, making these water-soluble extracts of plant material resemble cow’s milk in consistency, density, and overall appearance. From a physical and chemical point of view, plant-based beverages are colloidal suspensions or emulsions of selected plant constituents and/or their derivatives in water. Plant-based beverages are characterized as oil-in-water emulsions, where the oil is the dispersed phase in the aqueous phase. This type of emulsion allows it to mimic some of the characteristics found in milk, which is also an oil-in-water emulsion. These characteristics include appearance, consistency, stability, mouthfeel, and taste (sensory experience). The emulsifying substances in cow’s milk are phospholipids and milk proteins. In the case of plant-based beverages, these substances can be biosurfactants, phospholipids, proteins, and polysaccharides, which naturally occur in plant raw materials or are added during production [3,8,9].

#### 1.1.2. Classification and Characteristics of Raw Materials Used in the Production of Plant-Based Beverages

One of the main factors determining the composition and nutritional value of plant-based beverages is the type of raw material used in their production (Table 1).

## 2. Production of Plant-Based Beverages

### 2.1. Technological Process

**Figure 1:**
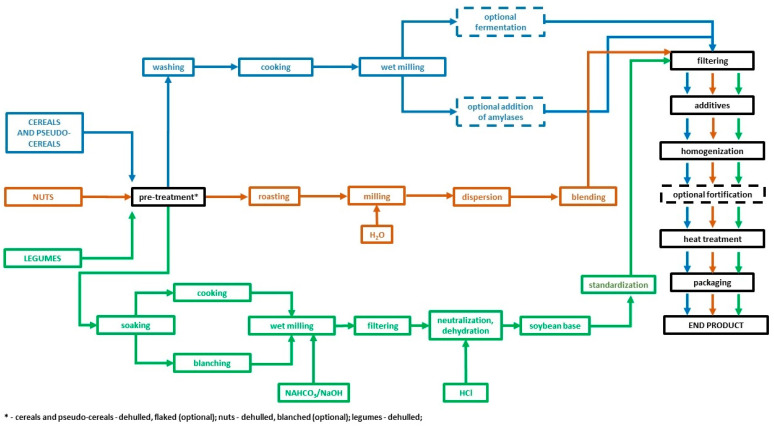
A simplified scheme for the production of plant-based beverages [4,6,9,10,15]. Blue colour—technological processes in the production of plant-based beverages typical of cereals and pseudo-cereals; Orange colour—vegetable beverage processes typical of nuts; Green colour—technological processes in the production of vegetable beverages typical of leguminous plants; Black colour—technological processes in the production of vegetable beverages involving the processing of vegetable beverages, irrespective of the raw material from which they are derived; Dashed line—optional technological processes.

The production of plant-based beverages varies depending on the raw material from which they are derived. The process involves many steps (Figure 1), including soaking, boiling/steaming to bind the starch, grinding, blanching (enzyme inactivation), centrifugation, heat treatment to ensure microbiological safety, homogenization, and, optionally, fortification with selected nutrients [10,11].

The soaking of legumes, hazelnuts, almonds, and chufa, as well as some cereals and sesame seeds, promotes the swelling and softening of seeds, kernels, and nuts, which reduces the apparent amylose content [5,12].

Blanching, used for example for soybeans, peanuts, almonds, coconuts, sesame, rice, and quinoa [5], is a thermal treatment that ensures microbiological safety and results in the inactivation of enzymes, such as lipases [13,14].

Wet grinding allows the raw material to be crushed, while factors such as the amount of water added, grinding temperature, pH, rotation speed, and grinding pressure and duration affect the extraction and quality of ingredients obtained from plant raw materials [6].

Water-insoluble solids are separated by filtration and/or ultrafiltration [5]. Processes such as high-pressure homogenization, ultrasound, enzymatic processes, and fermentation are increasingly being used in the production of plant-based beverages, which can increase yields and reduce waste [15].

Extended mechanical and thermal processes (e.g., roasting, dehulling, blanching, soaking, boiling, and sprouting) are used to reduce antinutrients such as protease inhibitors [16], to mask off-flavors, and improve consistency and color [17]. Some antinutrients are quite resistant to high temperatures. For example, phytates from cereals, pseudo-cereals, or legumes are not completely deactivated even by heating to 100 °C [18].

Fermented beverages are another direction in the development of plant-based beverages. Plant-based milk alternatives, by design, should resemble animal milk in color and consistency but may have an undesirable, unpleasant off-flavor [16]. One way to improve sensory quality is to use the fermentation process. Recently, mixed fermentation, which involves two or more microorganisms that occur naturally in many food production processes, has become increasingly important [19,20,21]. This offers the possibility of fermenting plant-based beverages, where the potential action of consortia of different microbial species can improve diverse quality criteria in a single process [22]. The most common microorganisms used in the fermentation of plant-based beverages are lactic acid bacteria, bacilli, and yeasts (e.g., *Saccharomyces*) [7], with a limited use of genetically modified lactic acid fermentation bacteria, characterized by the ability to synthesize amylolytic enzymes [23,24].

It should be noted that the production of food, including plant-based beverages, under organic farming certification requires that 95% of the raw materials from which plant-based beverages are obtained come from certified crops. According to European Union (EU) regulations (Regulation (EU) 2021/1165; Regulation (EU) 2018/848; OJ C 278, 12.07.2021) [25,26,27], organic methods of processing raw materials and obtaining plant-based beverages limit the application of:

- Some technological processes (e.g., sterilization);
- Some additives (e.g., carrageenan);
- Nutritional enhancers (e.g., using calcium carbonate to enrich products with calcium).

Fortification of organic products with vitamins and minerals is possible as long as their use is required in foodstuffs (e.g., they are included in foodstuffs for special nutritional purposes and can only be used to the extent that they are approved for use in organic production).

### 2.2. Additives and Technological Substances Used in the Production of Plant-Based Beverages

In addition to the basic ingredients, additives are used in the production of plant-based beverages (Table 2). Additives play an important role in providing the desired organoleptic characteristics and technological properties. Mixing two or more plant raw materials to produce a plant-based beverage affects changes in the nutritional value while providing a new product with an altered sensory quality.

### 2.3. Stabilizers and Thickeners

Hydrocolloids and/or dietary fiber are added to plant-based beverages to ensure stable, concentrated, uniform, smooth, and creamy consistency, which allows for the suspension of glucose starch molecules and extracted, homogenized plant components in a more viscous aqueous solution, thereby reducing the sedimentation effect. By prolonging stability, these properties help maintain the quality of plant-based beverages during storage.

Depending on the concentration, the pH of the environment, the presence of calcium, sodium, and potassium ions, and the concentration of sugars and salts in solution, hydrocolloids exhibit thickening, stabilizing, and gelling properties. The most common substances used in plant-based beverages are hydrocolloids such as locust bean gum, gums (gellan, xanthan, and acacia), and carrageenan, as well as dietary fiber fractions (pectin and inulin) and starches (tapioca and corn) [28]. In addition, when combined with proteins, hydrocolloids can produce a foaming effect and influence the stability of the resulting foam.

### 2.4. Emulsifiers

In plant-based beverages, emulsifiers that are commonly used in the food industry are utilized, such as lecithin and mono- and diglycerides of fatty acids. The addition of emulsifiers improves the oral texture perception and creaminess of beverages. Lecithin facilitates air bubble insertion and foam stabilization and can reduce fatty acid oxidation [29,30].

Proteins derived from plant raw materials (soybeans, peas, and lupins) also exhibit emulsifying properties but they can be sensitive to the pH of the plant-based beverage or to heat treatment, where the emulsifying ability is primarily affected by an altered charge distribution in protein molecules (tendency to denaturation or dissociation).

### 2.5. Acidity Regulators

In most plant-based beverages, the acidity regulator is tripotassium phosphate, which also exhibits buffer functions. Tripotassium phosphate forms complexes with multivalent metal ions, which can have pro-oxidant properties, stabilize the color of beverages, and promote emulsifying and dispersing activity [31]. According to Annexes II and III of Regulation (EC) No. 1333/2008 [32], phosphates are an authorized group of food additives in the EU countries.

### 2.6. Colorings and Flavorings

Palatability, which includes taste, smell, aroma, the oral perception of consistency and texture, and the overall olfactory response to ingested food, is an important characteristic of a plant-based beverage that determines its acceptance by consumers [33].

Plant-based beverages are fortified with natural colorings and flavorings (e.g., carrot extract, turmeric, cocoa, vanilla), which probably do not raise health concerns about their consumption. However, their structure can be easily degraded during technological processing (especially thermal processing) and storage [11].

### 2.7. Salt (Sodium Chloride)

Salt added to plant-based beverages is responsible for shaping flavor and influences the microbiological stability of the product by reducing water activity. The salt content in 100 mL of a plant-based beverage ranges from 0.08 to 1.6 g [27,33].

## 3. Nutritional Value of Plant-Based Beverages

### 3.1. Energy and Macronutrient Content in Plant-Based Beverages

The nutritional value of plant-based beverages is influenced by a variety of factors, including the type and quantity of base ingredients, the technological process, and the additives used in their production. It should be noted that the nutritional value of plant-based beverages differs from that of the raw material used in their production; therefore, it is important to carefully read the information provided on the packaging by the manufacturer, who is obliged to inform the consumer about the nutritional content of the product (Table 3 and Table 4). A wide range of plant-based beverages makes it possible to choose products tailored to individual consumer preferences and needs.

#### 3.1.1. Carbohydrates

The carbohydrate content of plant-based beverages is the sum of naturally occurring carbohydrates and those derived from the raw material(s), as well as added carbohydrates. It is generally higher than in cow’s milk (Table 3) and results from the addition of sweeteners and sugars such as sucrose, fructose, grape syrup (i.e., a mixture of glucose and fructose), and agave syrup (a rich source of fructose). Their role is to enhance the degree of sweet taste and act as one of the determinants of the microbiological quality of beverages [5,27,34].

#### 3.1.2. Protein

Plant-based beverages, except those based on soya beans and peas, contain significantly lower amounts of protein than cow’s milk (Table 3). However, the inclusion of plant-based beverages in the diet does not increase the risk of dietary protein deficiency [34,35]. The amino acid that limits the nutritional value of proteins is lysine in the case of cereals, and methionine and cysteine in the case of legumes [11]. In addition, the quality of plant proteins is slightly lower than that of animal proteins due to the presence of antinutritional factors, including phytic acid. The quality of protein in a single plant product may be low, but the quality of protein in a meal and in the overall diet can be increased by consuming a variety of foods in the same meal and throughout the day, which allows for replenishment of amino acids and improvement of protein quality. Therefore, it is important to provide amino acids from a variety of food sources throughout the day [36,37].

#### 3.1.3. Fats

Fats are considered one of the main carriers of flavor in products and foods. In plant-based beverages, they can be obtained from primary raw materials (e.g., soy, nuts, chufa) or can be added during production at the stage of mixing the ingredients and before their homogenization and heat treatment. The fat content of plant-based beverages ranges from only 0.4 g/100 mL to 19 g/100 mL (Table 3). Plant-derived fats (mainly sunflower and rapeseed oil) are added to plant-based beverages to improve texture and create an emulsion in water. This creates a smooth and creamy consistency in the mouth and shapes the smell and taste of plant-based beverages.

In contrast, the main cause of unpleasant taste in plant-based beverages is the oxidative degradation of fats. If beverages are stored under the conditions specified by the manufacturer, the quality of the beverage is relatively stable. However, endogenous lipoxidases can be activated during processing, leading to rancidity and the formation of undesirable off-flavors [32].

### 3.2. Micronutrient Content of Plant-Based Beverages

Plant-based beverages are naturally low in vitamin D. However, according to Crieg et al. [36] over three-quarters of plant-based beverages currently available on the European market are fortified with vitamin D_2_. If plant-based beverages are fortified with fat-soluble vitamins, the vitamin content can equal or exceed that of cow’s milk [38]. Because vitamin D facilitates calcium absorption and increases its bioavailability, plant-based beverages are usually fortified with both calcium and vitamin D [39].

Soy and almond beverages naturally have a higher vitamin E content than cow’s milk or can be fortified with vitamin, while coconut and almond beverages also have a higher provitamin A content (Table 4).

**Table 4:** Vitamin and mineral content in cow’s milk and selected plant-based beverages (with and/or without added sugar).

Nutritional Ingredients	Cow’s Milk *	Plant-Based Beverages **				
		Oat *n* = 80	Almond *n* = 50	Soy *n* = 100	Rise *n* = 50	Coconut *n* = 20
**Vitamins [100 mL]**						
A ^a^ [µg]	10.4 ^b^–61.6 ^c^	0–120	0–120	0–120	0–208	0–100
D [µg]	0.1–1.3	0–1.2	0–1.5	0–1.5	0–0.9	0–1.2
E [mg]	0.01–0.07	0–1.8	0–3.1	0–1.8	-	0–3.6
K [µg]	0–0.3	-	-	-	0–0.2	-
B_12_ [µg]	0.37–0.54	0–1.25	0–0.38	0–0.38	0–0.67	0–0.38
B_1_ *** [mg]	0.036–0.037	-	0–0.06	0–0.12	0–0.03	-
B_2_ *** [mg]	0.135–0.170	0–0.21	0–0.21	0–0.42	0–0.5	0–0.21
**Minerals [100 mL]**						
Calcium [mg]	113–134	0–128	0–120	0–144	0–120	0–170
Sodium [mg]	34.6–50.4	0.008–0.1	0.00–0.12	0.04–0.64	0.01–0.08	0.02–0.52

*n*—number of plant-based beverages available on the Polish market *—Antunes et al. [33]; **—data based on labels of selected plant-based drinks available on the Polish market; ***—Kunachowicz et al. [40]; ^a^—retinol equivalents; ^b^—minimum; ^c^—maximum.

The content of water-soluble vitamins in plant-based beverages may be insufficient unless they are fortified [11,34]. The production of plant-based beverages derived from cereals and pseudo-cereals can cause large losses of group B vitamins compared with the source material. Therefore, plant-based beverages are often fortified with thiamine (e.g., selected rice beverages) and riboflavin (e.g., selected beverages made from oats, spelt, almonds, nuts, soybeans, and peas).

Plant-based beverages are not a natural source of vitamin B_12_. However, as with other nutrients, vitamin B_12_ may be added during the fortification process. Therefore, B_12_-fortified plant-based beverages are important sources of dietary vitamin B_12_ for consumers following a plant-based diet [41].

Despite fortification, plant-based beverages are an insufficient source of minerals. Escobar-Sáez et al. [42] indicated that no plant-based beverage is a source of iodine and zinc, although seaweed-infused products that may contain iodine have appeared on the market. However, studies evaluating the iodine content of seaweed-infused beverages and iodine bioavailability are lacking. As for magnesium, phosphorus, and potassium, all plant-based beverages are lower in these minerals than cow’s milk, but unfortified soy beverages are only slightly poorer in these minerals than milk [32,43]. Because of these differences, the need to standardize the nutritional value of plant-based beverages is increasingly emphasized [34,44,45].

Some raw materials used in the production of plant-based beverages, including almonds and soybeans are naturally rich in calcium. Some calcium is lost during production (e.g., adding water and grinding). Therefore, calcium content in the final product depends on the proportion of raw material used and the production process (Table 4).

The bioavailability of calcium from foods, including plant-based beverages, depends on several factors, such as the form in which it is added or occurs naturally in foods, and the presence of ingredients that increase or decrease its absorption. Ruminant milk is characterized by a high content of calcium, which is highly bioavailable in the conditions of the gastrointestinal tract, thanks in part to an adequate calcium-to-phosphorus ratio (close to unity). In plant foods, phytates and oxalates form complexes with calcium that do not solubilize and ionize even at the low pH found during passage through the stomach, limiting the absorption of this component [41,46].

Calcium is a nutrient most often added to plant-based beverages to mimic cow’s milk as the main source of this mineral. Calcium fortification strategies should consider factors such as the target calcium content per serving of the product and the chemical form, which may determine its bioavailability. Shkembi and Huppertz [46] found that calcium absorption from soy drinks fortified with calcium carbonate (E 170) was similar to that from cow’s milk, and both were significantly higher than that from the soy drink fortified with tricalcium phosphate, with the same calcium load.

There is currently a trend to fortify foods with calcium derived from seaweed (*Lithothamnium calcareum*), although there is insufficient evidence to support this strategy (one study showed similar bioavailability to calcium carbonate [CaCO_3_] in piglets) [47].

The Panel on Food Additives and Flavorings of the European Food Safety Authority (EFSA FAF) [31] concluded that there is no need to establish a numerically acceptable daily intake of calcium carbonate and that there are generally no safety concerns regarding exposure to calcium carbonate at currently reported applications and use levels for all population groups.

The nutritional value of plant-based beverages is highly variable and, in many countries, largely unregulated. They do not have standards of identity. Therefore, Drewnowski et al. [48] developed standards for plant-based beverages’ energy content, minimum protein content and maximum content for added sugars, fat and salt (Table 5). They have also established a strategy for fortification with vitamins and minerals. However, these criteria are based primarily on plant-based beverages available in the US.

## 4. Potential Health Benefits and Risks of Consuming Plant-Based Beverages

### 4.1. Potential Benefits of Consuming Selected Ingredients with Plant-Based Beverages

Plant-based foods, including plant-based beverages, are growing in popularity, mainly due to concerns about our health and the health of our planet. Fortified plant-based beverages, produced by combining several raw materials, are recognized as alternatives (substitutes) to cow’s milk for health reasons, such as lactose intolerance, cow’s milk protein allergy, hypercholesterolemia, or for ethical and environmental reasons (Table 6).

Plant-based beverages can be considered part of a sustainable food system because they require less resource use (e.g., water and land use) and generate a lower carbon footprint compared with cow’s milk production. For example, it is estimated that the average carbon footprint of milk production is about 3.2 kg of CO_2_ equivalent per liter of milk produced, while the carbon footprint of various plant-based beverages ranges from 0.7 to 1.2 kg of CO_2_ equivalent per liter of beverage produced [49,50].

The nutritional value of plant-based beverages varies depending on the raw material and the technological processes used. Plant-based beverages made from legumes (soybeans, peas) have a relatively high protein content with a good amino acid composition. In addition, legumes and other seeds of dicotyledonous plants are rich in pectin and xylans, while seeds of cereals and other monocotyledonous plants are poor in pectin but contain arabinoxylan and/or beta-glucan. Oats are particularly rich in beta-glucan, which is a soluble fiber that increases solution viscosity. Beta-glucan can delay gastric emptying time and prolong gastrointestinal transit time, which is associated with lower blood glucose levels and has been shown to contribute to hypocholesterolemia. Almonds, on the other hand, are rich in alpha-tocopherol and manganese. Alpha-tocopherol is a functionally active component of vitamin E and is a potent antioxidant [4,14,37].

Plant-based beverages may also contain beneficial bioactive compounds, including flavonoids, phenolic acids, lignans, and phytosterols (Table 6). The role of flavonoids in plant-based beverages depends on their natural occurrence in plant raw materials or their incorporation during technological processes. Some plant-based beverages, such as those made from almonds and soy, may contain natural flavonoids derived from their primary ingredients, giving these beverages antioxidant properties. Therefore, people at high cardiovascular risk may experience increased benefits from consuming plant-based beverages rich in polyphenols. However, it should be noted that plant-based beverages available on the market are not typically fortified with flavonoids or polyphenols because the focus is mainly on vitamins and minerals to mimic the nutritional profile of cow’s milk [11,14,37].

Fortified plant-based beverages generally provide comparable amounts of calcium and vitamin D to cow’s milk, at similar levels of intake (Table 6). Hence, it is estimated that replacing one serving of cow’s milk with one serving of a fortified plant-based beverage (e.g., oat-based) in the Planetary Health Diet EAT Lancet model provides similar amounts of nutrients, especially calcium, while reducing the negative environmental impacts. Indeed, consuming one glass of soy or oat drink instead of a glass of cow’s milk reduces dietary greenhouse gas emissions by 9% or 12%, respectively [37].

However, because plant-based beverages as an alternative to dairy products are a relatively new concept, there are far fewer long-term studies available on their health effects than there are on milk and dairy products. The exception is soy drinks and soy products, which have a very long tradition of consumption in South and Southeast Asia. Thus, epidemiologic data on the consumption of these products and their health effects are more widely available, including for Western populations [51].

As mentioned above, the health effects of consuming plant-based beverages are often described as resulting from the effects and activity of the bioactive compounds present in the source material. Biscotii et al. [52], in a review of studies on the effects of plant-based beverages on health-related indicators, found that only a small number of human studies were available and that they varied in terms of participant characteristics, duration, and parameters assessed. It should be noted that mainly soy beverages were evaluated, with single studies on rice and almond beverages. Some of the studies reported beneficial effects of plant-based (soy) beverages on lipid profile and blood pressure. At the same time, it was not possible to draw any general conclusions due to some contradictions regarding the effects of these beverages on other health parameters, such as anthropometric measures, inflammatory and oxidative stress markers, glucose and insulin levels, or bone health markers. In addition, it should be noted that there are many more types of plant-based beverages available on the market (including spelt, oat, millet, buckwheat, nut, potato, etc.) than the three mentioned above, making it even more difficult to reach conclusions.

### 4.2. Potential Health Risks of Consuming Plant-Based Beverages

In terms of safety, several risks are associated with the consumption of plant-based beverages related to the raw materials used in their production. These risks include the ingestion of herbicides (particularly glyphosate and aminomethylphosphonic acid [AMPA]), arsenic, and mycotoxins; the risks associated with the use of genetically modified organisms (GMOs); and the potential allergenic effects of selected ingredients.

#### 4.2.1. Antinutrients

Plant raw materials contain antinutrients (Table 7), which can pass into plant-based beverages and contribute to various adverse effects, including reduced absorption of selected nutrients. Importantly, some steps of the production process (Figure 1), (i.e., soaking, heat treatment) can eliminate or reduce the amount of antinutrients in beverages and reduce their potential antinutritional properties. Information on antinutritional content is not provided on the label, so consumers are not informed about their impact on health [9].

Almonds, cashew nuts, and other nuts contain significant amounts of oxalates, which leach into beverages and inhibit calcium absorption. In addition, they can form a complex with calcium and contribute to the formation of kidney stones [53].

Phytates and saponins form insoluble complexes with minerals (such as Ca^2+^, Mg^2+^, Fe^2+^ and Zn^2+^), which reduces their bioavailability [54]. Saponins (Table 7) are glucosides that can be considered antinutrients because they interfere with the digestion of proteins, especially soy proteins, forming insoluble saponin–protein complexes that are resistant to digestion. They can also exhibit hemolytic effects. Different seeds, such as soybeans and oats, contain different saponins. However, in the processing of some plant-based beverages (such as soy beverages), heat treatment can reduce the content of these compounds [41].

Other antinutrients found in plant-based beverages include lectins (Table 7), which are commonly found in soybeans, peanuts, and other grains. They significantly inhibit intestinal glucose absorption and affect the use of energy from food [55]. Lectins and hemagglutinins are proteins or glycoproteins that contain at least one noncatalytic domain, thus exhibiting reversible binding to, in particular, monosaccharides or oligosaccharides. They can also bind carbohydrate residues present on the surface of erythrocytes, leading to erythrocyte agglutination [18]. Protease inhibitors (Table 7) are commonly found in plants and are becoming increasingly important in research as they strongly reduce enzyme activities by creating protein–protein interactions. Trypsin and chymotrypsin inhibitors are two types of protease inhibitors found primarily in legumes. They hinder the activity of trypsin and chymotrypsin enzymes in the intestine, resulting in the inhibition of protein digestion [56]. Cereal seeds primarily contain plant serpins, known as a family of major protease inhibitors. Serpins act as potent inhibitors of trypsin and chymotrypsin.

#### 4.2.2. Herbicides

The 2023 EFSA report [57] indicated that the sum of glyphosate and AMPA in plant-based foods was lower than 0.3–0.8 µg/L. Walther et al. [39] determined the content of selected pesticides in plant-based beverages, and the concentrations of glyphosate and AMPA ranged from 0.1 to 0.8 ng/mL. The concentrations were well below the acceptable daily intake and the acute reference dose (0.5 mg/kg bw/day) in the possible consumption scenarios.

In crop plants, tolerance to glyphosate is achieved by introducing a gene encoding the expression of a modified EPSPS (5-enolpyruvate-3-phosphate synthase) enzyme, making the plant insensitive to glyphosate.

Products derived from GMOs resistant to glyphosate are available on the European market [58]. The European Commission’s authorization for soybeans applies only to food use (e.g., the production of plant-based beverages) and does not allow its cultivation in the EU (Commission Implementing Decision, 2023/1207) [59]. At the same time, the use of GMO plants and their derivatives in food production should be indicated by the manufacturer on the product labelling. According to Article 13 of Regulation 1829/2003 [60], the label of a food product that contains or consists of GMOs, or is produced or contains ingredients produced from GMOs, should include one of the following indications: “genetically modified”, “produced from genetically modified (name of ingredient)”, “contains genetically modified (name of organism)”, “contains (name of ingredient) produced from genetically modified (name of organism)”.

Products containing GMOs at a level of up to 0.9% (of ingredients considered separately or of a single ingredient) are exempt from the labelling requirement, provided that the presence is unintentional or technically unavoidable (otherwise labelling is mandatory). The labelling requirement for GMO foods is intended to ensure that consumers can make an informed choice between genetically modified foods and their conventional counterparts.

#### 4.2.3. Arsenic

Arsenic is a metalloid commonly found in the environment due to both natural occurrence and anthropogenic activities. Arsenic occurs in various organic and inorganic forms. Guillod-Magnin et al. [61] indicated that arsenic content in rice beverages ranged from 10.2 to 12.4 µg/kg. Nearly 80% of total arsenic is present in the toxic inorganic form. It was reported that the consumption of three servings of a rice drink per day (0.6 L) would correspond to an intake of approximately 6.5 µg of inorganic arsenic (based on a mean arsenic concentration of 10.92 µg/kg in the five rice drinks tested) [61]. This level of exposure to inorganic arsenic is not insignificant, as the average intake for the Swiss population was estimated to be 0.029 µg/kg body weight per day for adults and 0.044 µg/kg body weight for children aged 1 to 3 years [39,61]. Although the benchmark dose lower confidence limit was defined at only 0.3 μg/kg body weight per day, arsenic exposure should be kept as low as possible [62]. It should be noted that the levels of arsenic in plant-based beverages were within the maximum range for drinking water in the European Union (Directive (EU) 2020/2184 of the European Parliament) [63]. In addition, the arsenic content of red seaweeds used for calcium fortification should be monitored due to the possible accumulation of arsenic [64]. At the same time, it is emphasized that the regular consumption of rice-based beverages can significantly contribute to arsenic exposure in the general population, which is why the British Food Standards Agency recommends that children under the age of 5 should not consume rice-based beverages.

#### 4.2.4. Microbiological Contamination and Presence of Mycotoxins

As with milk and dairy products, the process of obtaining plant-based milk alternatives includes high-temperature treatment (pasteurization, sterilization) to eliminate food-borne pathogens and spoilage-causing microorganisms [65]. Commercially available plant-based beverages are heat-treated to a point where they are considered microbiologically safe. However, there is still some risk of contamination of the finished product [66]. Despite the use of thermal processes, it was shown that under experimental conditions pathogens such as *Listeria monocytogenes* and *Salmonella* spp. were capable of growth and proliferation in plant-based beverages at a temperature of 4 °C, 8 °C, and 20 °C [67].

From a microbiological point of view, plant-based beverages can promote bacterial growth. They contain free monosaccharide sugars, such as fructose and glucose, which are metabolized by a number of different microbial species and can lead to a strong increase in bacterial load associated with sensory loss or, under certain circumstances, to cases of foodborne illness [66].

Spore-forming bacteria such as *Bacillus* and *Clostridium* are particularly difficult to remove from food ingredients or in food production facilities. A case of botulism associated with almond beverage consumption [68] highlights the need for increased awareness of the food safety risks posed by *C. botulinum*.

Mycotoxins are secondary metabolites of fungi that can be hazardous to human and animal health. The most common are aflatoxins, trichothecenes, fumonisins B_1_ and B_2_, zearalenone, and ochratoxin A [69,70]. Raw agricultural products, including cereals, legumes, and nuts, can be contaminated with filamentous fungi, leading to the risk of mycotoxins in processed foods such as plant-based beverages [71]. Mycotoxins exhibit acute and chronic toxicity, including genotoxic, carcinogenic, immunotoxic, mutagenic, nephrotoxic, and teratogenic effects [69,70]. They are harmful to health when consumed in significant amounts or on a continuous basis [72].

Numerous studies have assessed exposure to mycotoxins from the consumption of teas as well as fruit and vegetable juices. However, data on such exposure from the consumption of plant-based beverages are still lacking. Based on the assumption that plant-based milk substitutes are most commonly used as an addition to tea and coffee, Pavlenko et al. [72] calculated the probable daily intake (PDI) values (2024). The PDI values for the average levels of mycotoxin contamination ranged from 0.01 to 7.62 ng kg^−1^ body weight per day for all age groups of men, while the highest levels were reported for the exposure of men aged 19 to 39 to nivalenol. The lowest value was determined for aflatoxin B1 based on average contamination levels in nut-based beverages. For women, the PDI for the average contamination levels ranged from 0.01 to 4.29 ng kg^−1^ body weight per day, and the highest levels were found in women aged 19 to 34 years for exposure to nivalenol. Simulations of PDI results for Northern European adults showed that the average PDI concentrations for ochratoxin A, deoxynivalenol, and zearalenone were 0.6764, 0.48282, and 0.00149 µg/kg body weight per day, respectively [72]. Hence, it can be assumed that the levels of mycotoxin contamination in plant-based milk substitutes are very low compared with these simulated values. Overall, based on the average upper-bound contamination levels, Pavlenko et al. [72] concluded that all the plant-based beverages tested were safe for human consumption.

#### 4.2.5. Carrageenan

Similar to other additives, the quantitative contribution of added carrageenan in plant-based beverages is not declared by the manufacturer on the label, but for technological reasons, it must not exceed acceptable quantum satis levels [73]. According to the EFSA, the existing acceptable daily intake for carrageenan (E 407) and processed *Eucheuma* seaweed (E 407a) at a dose of 75 mg/kg body weight per day should be considered provisional [74].

Low-molecular-weight carrageenan (thickeners) is suspected of causing inflammation and precancerous changes in the intestines [75]. In vitro and animal studies suggested proinflammatory effects of carrageenan. However, it is not possible to translate the results obtained in vitro and/or in animal models to humans, although a cellular model has been tested and shown to have similar functional mechanisms. Data on dietary carrageenan intake and its content in various products and diets are lacking. Until such data are available, it is advisable to limit human exposure to carrageenan by reducing its use in foods [76].

#### 4.2.6. Selected Allergens in Plant-Based Beverages

Caution should be exercised when serving plant-based beverages to individuals with suspected and/or known allergies to proteins of tree nuts and peanuts, cereals, and legumes such as soybeans and lupins.

Legumes are an important source of protein in a vegan diet. At the same time, they are known to cause allergic reactions. The prevalence of allergy to legume proteins depends on several factors, including geographic region and dietary habits. For example, allergy to lentil proteins is more common in Mediterranean countries, and to chickpea proteins in India [77].

Soy allergy is mainly caused by storage proteins such as 11S glycine or 7S ß-conglycinin (Gly m 5), which are associated with severe allergic reactions [78]. The PR-10 protein Gly m 4 can cause oral allergy syndrome in people allergic to birch pollen, including severe anaphylactic reactions in the presence of cofactors or after ingestion of soy drinks. This is due, on the one hand, to the high incidence of hypersensitivity to PR-10 and, on the other hand, to the increasing use of soy-based beverages as an alternative to cow’s milk [79]. Heat treatment does not reduce the allergenic potential of soy-based beverages.

Lupin is used as an alternative to soy in baked goods, pasta, and plant-based beverages. Currently, three allergens are available for diagnostic purposes: the major allergen Lup an 1 (a storage protein), Lup an 3 (a lipid transfer protein), and Lup an 5 (from the profilin family). In addition, three low-molecular-weight proteins (Lup a alpha-, Lup a gamma-, and Lup a delta-conglutinin) were identified, as well as two proteins from the PR-10 family. Lupin was initially described as causing cross-reactivity with peanuts, possibly mediated by Ara h 1 and Lup an 1. Cross-reactivity with peas and lentils was also reported [79].

There are 18 peanut allergens registered in the allergen database of the WHO/IUIS Subcommittee on Allergen Nomenclature [80], with the most important allergens appearing to be the seed storage proteins Ara h 1, Ara h 2, and Ara h 3. In addition, allergens from these three families have been identified as major allergens in other legumes and tree nuts, so frequent cross-reactions are observed in people who are allergic to peanuts, other legumes, and tree nuts.

Amandine is the main protein responsible for allergies to almonds. Devnani et al. [81] and Dhakal et al. [82] examined the effects of heat and high-pressure processing in almond beverages and found lower protein levels, including amandine. However, research showed the presence of heat-stable allergens that can trigger allergy symptoms [83]; therefore, individuals with an allergy to almonds should not consume products made from or containing almonds.

It is increasingly common to enrich plant-based beverages with plant protein concentrates or isolates to balance amino acid quality. For example, pea protein is often used, which poses a challenge in terms of allergen labelling for those allergic to this protein. This is because peas and pea-derived products are not currently subject to mandatory labelling under Reg. 1169/2011 [84]. In addition, the main allergenic pea proteins are heat-resistant, and the cooking process does not affect allergenicity. Seven allergens found in peas have been described: Pis s 1, Pis s 2 (storage proteins), Pis s 3 (lipid transport protein), Pis s 5 (profilin), Bet v1 homolog Pis s 6 (PR-10 protein), as well as agglutinin and albumin. Cross-reactivity with peanuts, lentils, and chickpeas was reported [79].

Another difficulty for consumers is the risk of resulting cross-reactions. This is particularly important for families who choose plant-based beverages due to the presence of food allergies to animal products (mainly cow’s milk proteins).

## 5. Labeling of Plant-Based Beverages

According to Part E of Annex II to Regulation (EC) No 1333/2008 [32], plant-based beverages are included in the non-alcoholic beverages group and today, consumers are informed about the characteristics of a food product through food labelling and advertising in accordance with EU Regulation 1169/2011 [84], and especially through the nutrition and health claims contained therein [85]. Although they are not mandatory information on food labels, if they are on the package, they should be clear, accurate, and based on scientific evidence. Consumers tend to perceive products with claims on the label as “more” beneficial to health than those without claims, and often attribute more nutritional and/or functional benefits to them than the label claims imply [86].

Plant-based beverages used as an alternative to milk were customarily referred to as plant-based “milks”. This term has been considered incorrect and misleading to consumers in Europe [87]. However, a decision by the European Union Commission [88] allows some exceptions to the use of the term “milk” for certain non-dairy plant-based beverages that are traditionally called (e.g., “almond milk” in Spain or “coconut milk” in Portugal). In other countries, the term “mylk” (modified from “milk”) is used for plant-based beverages due to pressure from the dairy industry to differentiate products [89]. Despite the outcry against this exclusive designation of milk, the definition was confirmed by a ruling of the Court of Justice of the EU on 14 June 2017 [90].

It should be remembered that milk is the liquid secretion of the mammary glands of female mammals, and this name is reserved exclusively for the udder secretions of cows, which must not contain any additives and must not be subjected to extraction. In the case of udder secretions from the udder of other domesticated mammals, the name should be expanded to include the species of animal from which the secretion comes, such as goat milk, donkey milk, or camel milk [90].

Attention should be paid to the obligation to label foods, including plant-based beverages, with allergen information [84]. The list of substances and/or products and their derivatives that cause food allergies or intolerance reactions is presented in Annex II to Reg. 1169/2011 [84]. Examples of allergens found in plant-based beverages include cereals containing gluten, (i.e., wheat, spelt, barley, oats), tree nuts and peanuts, and soy-based products. The manufacturer is obliged to provide information on the label about the substance that causes allergies or intolerance reactions, and the name of the substance must be highlighted with a typeface that clearly distinguishes this information from the list of other ingredients in the finished product, for example, by style, size, or background color. Information to the consumer about allergens must be easily accessible, clearly visible, and legible.

## 6. Plant-Based Beverages in the Nutrition of Selected Population Groups

### 6.1. Use of Plant-Based Beverages in the Nutrition of Infants, Children, and Adolescents

#### 6.1.1. Infants (0–12 Months)

The goal of infant nutrition is to promote exclusive breastfeeding for the first 6 months of life, which should be continued for as long as desired by the mother and child. If infants are not breastfed, they should be given breastmilk substitutes. No plant-based beverages are recommended for this age group, as they do not provide the necessary nutrients in sufficient quantities.

According to the position of the Nutrition Committee of the North American Society for Pediatric Gastroenterology, Hepatology and Nutrition [91], the inappropriate inclusion of plant-based beverages in the diet of young children can lead to negative health consequences, such as inadequate weight and growth gain, risk of iron deficiency anemia, rickets, electrolyte disturbances, and kidney stones. The committee emphasized that only soy formulas for infants can be an alternative to breast milk in special situations. This position is shared by other scientific societies and expert groups, including the Polish Society of Gastroenterology, Hepatology and Child Nutrition [92].

#### 6.1.2. Soy Formulas for Infants

Soy formulas that meet the requirements for breastmilk substitutes can be used in infant nutrition [93,94]. In these formulas, the source of protein is isolated soy protein, fortified with L-methionine, L-carnitine, and taurine. Fats are derived from vegetable oils (such as soybean, sunflower, palm, safflower, coconut, and in some cases, olein) and carbohydrates from corn starch or its hydrolysis products (glucose polymers), tapioca starch, or sucrose. These formulas do not contain lactose. Soy infant formulas are fortified with minerals, but the content of many of them, especially phosphorus and calcium, is higher than in modified infant formula due to the presence of phytates, which inhibit phosphorus and calcium absorption [95].

Indications for the use of soy infant formulas are:

- Galactosemia;
- Congenital lactase deficiency;
- Documented secondary lactase deficiency;
- Religious, ethical, and philosophical considerations (e.g., vegetarian diet).

The role of soy formulas in the treatment of cow’s milk protein allergy remains debated (see below).

#### 6.1.3. Young Children (<3 Years of Age)

Young children (aged 1 year to 3 years) can be given whole cow’s milk or milk-based formulas fortified with vitamins and minerals. This ensures that the child’s diet contains adequate amounts of iron, calcium, iodine, vitamin D, and other essential vitamins and minerals.

In children of this age group, milk designed for young children is often used, known in Poland as “Junior type”. Both the EFSA and the European Society for Paediatric Gastroenterology, Hepatology and Nutrition (ESPGHAN) [96] recommend using the term “young child formula” (YCF). According to the ESPGHAN [96], it is not necessary to routinely give YCF to children aged >1–3 years. However, YCF can increase the intake of iron, vitamin D, and long-chain omega 3 polyunsaturated fatty acids, and reduce protein intake compared with cow’s milk [97,98,99,100]. When parents choose plant-based beverages for their children, it is important to choose fortified options [91,101].

Although there are no definitive guidelines, a group of experts recently identified key factors to consider when determining if a toddler is ready to transition to a commercial plant-based beverage [102]. The following aspects should be considered:

- The toddler is at least 12 months old;
- Their diet includes a variety of solid foods from all food groups;
- At least two-thirds of their daily energy intake comes from solid foods;
- They consume no more than 500 milliliters of milk substitutes per day, including breastmilk, formula, or other dairy alternatives such as yogurt;
- They can eat foods with consistencies suitable for their age;
- Their diet provides sufficient protein, fats, and essential micronutrients from solid foods and milk substitutes;
- They do not have feeding difficulties that limit the variety of foods they consume.
- They have no diagnosed micronutrient deficiencies;
- Their diet is not restricted by cultural or religious practices that reduce food variety;
- These criteria aim to ensure toddlers meet their nutritional needs during this dietary transition.

#### 6.1.4. Adolescents

The choice of plant-based beverages should be tailored to the overall diet and provide nutrients and can add variety to an adolescent’s daily diet. However, if used, fortified plant beverages should be considered due to the increased needs of young people during this period of development. Fortified plant-based drinks can add variety to the daily diet of teenagers. However, due to the increased need for energy, protein, calcium, and vitamin D at this age compared with younger children, special attention should be paid to the overall diet quality. It should be noted that in the long term, inadequate intake of nutrients can lead to nutritional deficiencies and diseases later in life in adolescents due to their rapid growth and development.

### 6.2. Plant-Based Beverages in the Nutrition of Women During Pregnancy and/or Lactation

Proper nutrition during pregnancy and lactation is essential for the health of the mother and her baby. An unbalanced diet during pregnancy and lactation is associated with negative health effects for the child. Therefore, the nutrition of women during pregnancy and lactation requires conscious and responsible nutritional management [99,103]

Alternative diets, including planetary, vegetarian, and vegan diets, are becoming increasingly popular, also among women during pregnancy and lactation. Expert opinions on the nutrition of pregnant and lactating women on various types of vegetarian diets emphasize the importance of foods with high nutritional value prepared from plants, including legumes such as peas, beans, soybeans, and nuts. These include fortified plant-based beverages, pasta, and plant-based alternatives to cheese and meat [104,105].

Plant-based beverages fortified with calcium and vitamin D can be an alternative source of potentially deficient nutrients, such as dairy-free diets for women during pregnancy and lactation. It is believed that at least one serving of the recommended three servings of products replacing milk and dairy products should be fortified with calcium (e.g., one glass of plant-based beverage providing at least 300 mg of calcium [120 mg/100 mL]) [98,103].

### 6.3. Plant-Based Beverages in the Nutrition of People Aged over 65 Years

Elderly people with chronic diseases or at risk of sarcopenia or osteoporosis require special nutritional attention. Older people who want to reduce or eliminate milk in favor of plant-based beverages are advised, similar to other adults, to choose beverages fortified with nutrients such as calcium and vitamins D and B_12_ [38,106]. In elderly people, it is important to ensure adequate calcium intake as calcium absorption becomes less efficient with age and bone density also declines with age. Therefore, calcium-fortified plant-based beverages can be an important source of calcium in their diet.

Protein intake is of particular concern in this age group. It is believed that the protein intake in individuals over the age of 65 should be higher to maintain muscle mass and prevent sarcopenia, and in the case of chronic or acute conditions [107,108,109]. The lower quality of protein available in some plant-based beverages should also be noted [38,43].

Absorption of vitamin B_12_ from food in the elderly may be impaired due to physiological changes in the gastrointestinal tract and impaired gastric secretions. Thus, vitamin B_12_ status in elderly persons can be improved by regular consumption of plant-based beverages fortified with this vitamin. Considering the above arguments, the most optimal choice of a plant-based beverage for this age group would be a fortified soy beverage because it has a higher protein content than the other beverages [38,43,110,111]. Other plant-based beverages, such as oatmeal, almond, and rice, have lower protein content compared with milk and soy beverages, and their consumption should be supplemented with other sources of dietary protein to prevent sarcopenia and loss of muscle mass.

Given the reduced thirst sensation in some elderly people and frequent lactose intolerance, fortified soy beverages can play a hydrating role. In contrast, in the prevention of overweight and obesity, attention should be paid to the sugar content of these beverages.

### 6.4. Plant-Based Beverages in the Nutrition of People Following Plant-Based Diets

In recent years, there has been a growing interest in and preference for plant-based diets among adults [112]. The motivation for individuals to adopt such diets has also changed recently. In addition to health aspects and those related to weight loss, environmental and animal welfare concerns are also increasingly reported reasons [113,114].

The adoption of a plant-based diet has been shown to be important for human and environmental health in developed countries. Products such as plant-based beverages can play an important role in this process and are part of sustainable dietary patterns. In addition, compared with traditional milk, they contain lower amounts of saturated fatty acids, which are detrimental to cardiovascular health [38].

In particular, soy beverages fortified with calcium are a valuable alternative to milk in terms of protein and calcium content and are recommended as equivalent to milk in dietary recommendations in other countries [115,116,117,118,119,120,121,122]. At the same time, attention should be paid to the variety and quality of other foods in the diet, with a particular focus on the sources of calcium, protein, and vitamin B_12_ to ensure that all the nutrients needed to maintain good health are provided [37].

Recommendations for the use of plant-based beverages should focus on the potentially deficient components and depend on the type of plant-based diet and its nutritional value [123]. From a nutritional point of view, replacing milk with plant-based beverages in people on plant-based diets should not pose a risk of protein deficiency, provided that they follow a well-balanced varied diet. It is recommended that a fortified soy beverage is the primary plant-based beverage in individuals who have difficulty obtaining adequate protein supply and that other adults on plant-based diets also use other fortified plant-based beverages.

## 7. Plant-Based Beverages in the Diet of People with Selected Health Problems

### 7.1. Cow’s Milk Protein Allergy

The basic step in the treatment of cow’s milk protein allergy (CMPA) is the use of an elimination diet, which excludes the consumption of cow’s milk and products containing cow’s milk proteins. According to the current ESPGHAN guidelines [124]:

- In formula-fed infants, a cow’s milk-derived extensively hydrolyzed formula is the first choice for a therapeutic elimination diet;
- In CMPA patients with severe diarrhea and/or significant malnutrition, short-term use of a lactose-free formula for 2–4 weeks may be considered;
- Amino acid formulas can be used as first-line therapy in selected severe cases of CMPA, including patients with an anaphylactic reaction, eosinophilic esophagitis, or infants who show partial or no response to an extensively hydrolyzed protein formula.

Rice protein hydrolysates or soy formulas are also used in the treatment of CMPA; however, scientific societies differ in their recommendations regarding their use, which are inconsistent. According to ESPGHAN [124] and the World Allergy Organization [125], rice protein hydrolysates can be used for treating CMPA. However, according to the GA2LEN guidelines [126], there are insufficient data to make a recommendation for or against the use of rice protein hydrolysates for the treatment of food allergy in infants [125].

According to the ESPGHAN [124], soy formulas (i.e., formulas in which the protein source is isolated soy protein) are not recommended as a first-line treatment for CMPA. They can be considered for use in children even under 6 months of age if taste or cost are important. In contrast, according to the GA2LEN guidelines [126], soy formulas should not be used in infants under 6 months of age.

### 7.2. Lactose Intolerance

Lactose (milk sugar) is a disaccharide composed of D-glucose and D-galactose molecules linked by a β-1,4-glycosidic bond. This bond is hydrolyzed by lactase, an enzyme produced by enterocytes located in the brush border of the small intestinal epithelium. Lactase activity decreases with age, leading to a progressive reduction in lactose tolerance.

Lactose intolerance is a clinical syndrome defined as the occurrence of gastrointestinal symptoms after ingestion of lactose by a person with lactose malabsorption syndrome. Congenital or acquired inability to produce lactase leads to impaired digestion and absorption of lactose, the appearance of larger amounts of undegraded lactose in the large intestine, which is a substrate for microbiota [127]. The products of bacterial fermentation cause undesirable health effects, such as bloating, abdominal pain, colic, overflow, irritation of the intestinal mucosa, and diarrhea [128,129].

The main treatment for lactose intolerance is to restrict lactose-containing foods and include lactose-free dairy products in the diet. Most people with lactose intolerance tolerate a single dose of 5 g to 12 g of lactose in a meal well but this is individual. Complete elimination of milk and dairy products is rarely necessary and can lead to nutrient deficiencies and the risk of osteoporosis [127].

Plant-based beverages are a natural, lactose-free alternative to dairy drinks (milk and fermented beverages) and can be safely included in the diet of people with lactose intolerance. When choosing plant-based beverages, products fortified with calcium and vitamins should be preferred. Given the lower bioavailability of calcium from plant-based beverages compared with lactose-free dairy drinks, it may be reasonable to include plant-based beverages fortified with calcium at an amount 20% higher than the dietary reference intake. Most plant-based beverages contain about 120 mg of calcium per 100 mL. Beverages containing 170–189 mg calcium/100 mL beverage are also available [130,131,132,133].

### 7.3. Celiac Disease

Celiac disease is a chronic, immune-mediated disorder in which the small intestine of genetically predisposed individuals is damaged by the ingestion of gluten [134]. The only effective treatment for celiac disease is a lifelong gluten-free diet. Products that are a source of gluten should be completely excluded from the diet of patients with celiac disease and replaced with gluten-free products, such as plant-based beverages made from legumes, pseudo-cereals, and tree nuts.

The consumer must be aware that the basis of the treatment process is to maintain a strict gluten-free diet and should bear in mind the following principles [134]:

- The manufacturer is obliged to label food products with information on the presence of gluten and gluten-containing cereals and their derivatives as required by Annex II of Regulation 1169/2011 [84];
- The need to pay attention to plant-based beverages with the following information on the packaging: “may contain gluten” or “gluten (or wheat) is used on the premises”, or “contains wheat”, and thus pose a health risk as the gluten content may be higher than 20 ppm;
- The need to regularly check the information on the labels, as the composition of the product may change, and a previously “safe” plant-based drink may turn out to be a serious health hazard;
- The need to regularly read the labels of all plant-based beverages, including those that are not associated with cereal content (e.g., soy, almond, buckwheat, etc.);
- Naturally gluten-free products (e.g., plant-based beverages made from maize, buckwheat, or soy) may become contaminated with gluten during their harvesting, processing, and packaging, so patients should be advised to buy the above products labelled as a “gluten-free product”, meaning that the gluten content does not exceed 20 mg/kg (20 ppm) in the food sold to the final consumer (Regulation (EC) 828/2014) [135].

Monitoring the nutritional value of plant-based beverages is important to adequately replace cow’s milk with plant-based substitutes in the diets of people suffering from celiac disease.

## 8. Recommended Approaches Regarding Introduction of Plant-Based Beverages

These beverages cater to a wide range of consumers, including children with cow’s milk protein allergies, adults, the elderly, and vegetarians. For children under the age of 3, PBBs should not be considered as dietary alternatives because they lack essential nutrients naturally found in cow’s milk or formulas (Table 8, Table 9 and Table 10). In adults and the elderly, these beverages provide dietary diversity and can align with health-conscious dietary choices, including plant-based diets. However, given their lower protein quality compared to dairy milk, individuals relying on PBBs as a primary milk alternative should ensure adequate protein intake from other sources.

Despite their advantages, the selection of plant-based beverages should be made carefully to ensure adequate intake of essential nutrients (Table 8, Table 9 and Table 10). Fortified options are recommended, especially for individuals at risk of deficiencies. A clear and accurate labeling system, along with proper consumer education, is essential to guide individuals in selecting nutritionally balanced PBBs. Studies highlight that misleading claims on packaging can contribute to misconceptions regarding the nutritional adequacy of these beverages. Future research should focus on enhancing their nutritional profile and improving digestibility to ensure they serve as balanced dietary alternatives. Improved fortification strategies should emphasize the use of bioavailable and soluble nutrient derivatives to maximize absorption. Additionally, novel plant sources, such as fruit kernels, are being explored to diversify PBB formulations and enhance their functional and nutritional value. The following recommendations are based mainly on expert opinions, which support the usefulness or effectiveness of a given procedure on the basis of objective results of scientific studies [93,96,97,100,101,109]. The level of data credibility is the B and C scale, i.e., data from individual studies or expert opinions.

## 9. Conclusions

Despite some similarities with cow’s milk, such as appearance and texture, plant-based beverages are characterized by diverse nutritional values, which largely depend on the raw material composition (plant matrix), mainly in terms of protein and amino acid content, as well as the technological process, especially in terms of vitamin and mineral fortification, and the fermentation process.

The choice of plant-based beverage will, therefore, depend on the purpose (nutritional or sensory) and the preferences or limitations (e.g., health) of the consumer. Fortified plant-based beverages can be a good addition to a well-balanced diet of healthy adults.

Due to differences in nutritional value and composition, most plant-based beverages cannot fully replace the cow’s milk matrix in terms of quality and nutritional value. Some of these beverages contain ingredients (legumes, almonds, nuts, seeds, etc.) that may also be allergenic to some people. In such cases, their use should be approached with great caution. In addition, differences in the quality (amino acid composition) and quantity of protein are particularly important for groups with increased protein requirements and/or low protein intake, such as the elderly, children, and people who consume no or very little animal products and whose diets are not properly balanced. On the other hand, the use of plant-based beverages that are not fortified with, for example, calcium, vitamins D, B_12_, and B_2_, as well as organic products that cannot be subjected to such processes, can lead to imbalanced diets and significant deficiencies and health consequences, including impaired growth, osteoporosis, and anemia.

There is a need for long-term studies evaluating the health effects of plant-based beverages and education on how to choose the best alternative to add variety to a balanced diet or to use them for health or ethical reasons.

Education of parents and caregivers on appropriate nutrition for infants, children, and adolescents is crucial to inform about the risks of using plant-based beverages as a substitute for breast milk or modified cow’s milk. Since the nutritional profile of plant-based beverages varies across different plant-based drink varieties and they do not have standards of identity, in our opinion, there is a need for action to standardize nutrient fortification regarding the type and amount of added ingredients to ensure the safety of consumers and avoid potential over- or under-fortification of plant-based beverages. Therefore, selecting new plant sources or the right combination of plant-based ingredients, optimizing the processing, and increasing the beneficial nutrients (i.e., iodine, magnesium and probiotics) while reducing nutrients of concern (i.e., added sugar, fat and sodium) appear to be future strategies for creating widely accepted, nutritionally complete, allergen-free, low-cost and safe plant-based beverages for consumers.

## Figures and Tables

**Table 1:** Plant raw materials used in the production of plant-based beverages (own elaboration).

Origin	Botanical Species
Cereals	ciborium (chufa, tiger nut), barley, maize, oats, millet, wheat (common, spelt, kamut), rice, sorghum
Pseudo-cereals	amaranth, buckwheat, quinoa, teff
Legumes	chickpeas, peas, lupins, peanuts, soybeans
Tree nuts	chestnut, coconut, macadamia, almond, cashew (Nanner nut), Brazil nut, hazelnut, pecan, walnut
Seeds	pumpkin, hemp, flax, sesame, sunflower
Tubers	potato

**Table 2:** Major ingredients and additives used in the production of plant-based beverages [28].

Fats	Sweeteners and Sugars	Thickeners, Stabilizers, and Gelling Agents	Emulsifiers	Acidity Regulators	Food Color Additives and Flavorings
sunflower oil rice oil canola oil linseed oil hemp oil fully hydrogenated coconut fat ^a^	sucrose, cane sugar fructose grape juice or juice concentrate apple juice or juice concentrate agave syrup glucose syrup	locust bean gum carrageenan gellan gum xanthan gum inulin acacia gum pectin maize maltodextrin rice starch tapioca starch	soya lecithin mono- and diglycerides of fatty acids	tripotassium phosphate tricalcium phosphate	carrot extract ^b^ turmeric ^b^ cacao ^b,c^ coconut ^c^ vanilla ^b^ vanillin ^b^

^a^—mainly used for powdered plant-based drinks; ^b^—food color additive; ^c^—food flavor additive.

**Table 3:** Energy and macronutrient values of cow’s milk and plant-based beverages (with or without added sugar).

Nutritional Ingredients	Cow’s Milk *	Plant-Based Beverages *				
		Oat *n* = 80	Almond *n* = 50	Soy *n* = 100	Rise *n* = 50	Coconut *n* = 20
**Energy [100 mL]**						
kJ kcal	141 ^a^–295 ^b^ 33.0–70.0	132–331 31–79	48–380 12–91	78–321 18–76	85–340 20–80	63–764 15–185
**Carbohydrates [g/100 mL]**						
total	4.10–5.5	5.6–13.4	0–11.6	0–10	0.9–18	0.3–11.4
sugars	3.38–4.8 **	0–9.2	0–9.0	0–10.0	1.9–10.4	0–7.4
fiber	-	0–1.4	0.1–1.2	0.2–5.0	0–1.0	0–0.7
**Protein [g/100 mL]**						
total	3.20–8.00	0–3.2	0.3–1.8	1.8–5.0	0–3.7	0.1–1.6
**Fats [g/100 mL]**						
total	0.0–4.8	0.7–3.5	0.6–5.2	0.5–2.9	0.4–2.6	0.9–19
SFA	0.32–2.65	0–0.4	0.1–4.9	0.1–0.7	0–1.1	0.8–17
MUFA	0.12–1.11	0.1–0.9	0.6–1.4	0.41–0.10	0.1–4.7	0–1.1
PUFA	0.0–0.18	0.2–0.7	0.1–0.8	0.8–1.2	0.1–0.6	0–1.2

*n*—number of plant-based beverages available on the Polish market; *—data based on labels of selected plant-based beverages and cow’s milk available on the Polish market; **—lactose content in cow’s milk: 0.01 g/100 mL (in lactose-free milk) to 4.9 g/100 mL; ^a^—minimum; ^b^—maximum; SFA—saturated fatty acids; MUFA—monounsaturated fatty acids; PUFA—polyunsaturated fatty acids.

**Table 5:** Energy content and minimum nutrient standards for plant-based beverages adapted from Drewnowski et al. [48].

Nutrient	Proposed Nutrient Standards Per 100 g of Plant-Based Beverages	
	Children (4–12 years)	Adults (>12 years)
Energy [kcal]	<85	<100
Protein ^a^ [g]	>2.2	>2.2
Total/added/free sugar [g]	<5.3	<6.25
Saturated Fat [g]	<0.75	<0.75
Sodium [mg]	<120	<120
Calcium [mg]	>15% DV DV/200 g serving	>15% DV DV/200 g serving
Riboflavin [mg]	>15% DV DV/200 g serving	>15% DV DV/200 g serving
Vitamin D [IU]		
Vitamin B_12_ [mcg]		
Vitamin A ^b^ [mcg]		
Fiber [g]	Optional	Optional
Carbohydrates [g]		
Potassium [mg]		

DV—daily value; ^a^—protein quality (protein digestibility corrected amino acid score, PDCAAS) > 0.9 and >0.8, respectively; ^b^—retinol equivalents.

**Table 6:** Summary: milk vs. plant-based beverages [4,8,10,16,27,34,35,39,43,47].

Milk	Plant-Based Beverages
a complex colloidal dispersion (oil-in-water emulsion) high-quality protein, provides all the essential amino acids essential vitamins B_2_, B_12_ essential minerals (calcium, phosphorus, potassium and iodine) a naturally occurring sugar (lactose)	oil-in-water emulsions the lower protein content (except for soy beverages) lack certain essential amino acids artificially added calcium, rarely iodine very high variability of solubility and calcium bioavailability added sweeteners and sugars
the milk matrix calcium naturally associated with casein	a highly processed food isolated and individually added nutrients a high degree of variability in the nutrient composition
better stability of emulsion better bioavailability (e.g., calcium)	diverse nutritional values, which largely depend on the raw material composition (from plant matrix) sedimentation of added calcium undesired flavor
minimal processing steps (4–6 steps)	long processing steps (13–15 steps)
naturally occurring bioactive substances (e.g., bioactive peptides, α-lactalbumin, immunoglobulins, lactoferrin, growth factors, glycomacropeptide, milk fat globule membrane, and milk oligosaccharides) cow’s milk proteins may be a potential allergen potentially occurring contaminants (e.g., pesticides, veterinary medicines, heavy metals, mycotoxins)	naturally occurring bioactive substances (e.g., phytosteroles, β-glucan) the potential presence of anti-nutritional factors (e.g., oxalates, phytates, lectins, saponins which are bioactive components present in plants that impact absorption of nutrients) lower bioavailability of calcium, zinc and iron due to high levels of phytates, oxalates and tannins potentially toxic naturally occurring compounds (e.g., cannabinoids) greater allergenicity contaminants (e.g., As)

**Table 7:** Selected antinutrients found in raw materials used for plant-based beverage production [18].

Antinutrients	Origin	Potential Effect
Phytates and oxalates	seeds, nuts, cereals	reduced absorption and bioavailability of minerals
Lectins and hemagglutinins	wheat, beans, quinoa, peas, potato	inhibition of glucose absorption, reduced acquisition of energy from food
Trypsin inhibitors	soybeans, chickpeas	protein digestion disorders
Alpha-amylase inhibitors	pumpkin, sesame, sunflower	inhibition of salivary and pancreatic amylase activity
Protease inhibitors	cereals and legumes	reduced protein digestion
Saponins	pseudo-cereals, legumes, potato	bitter taste, reduction of bioavailability of minerals, interference with protein digestion and vitamin and mineral absorption in the intestines

**Table 8:** Recommended approaches regarding introduction of plant-based beverages in human nutrition.

Plant-Based Beverages		
Recommended	Caution	Not Recommended
as a part of varied diet of toddlers, adolescents, adults and older persons, foremost if they are fortified with vitamin B_12_ and calcium	in case of allergy to cow’s milk (except for infants and young children) can be a substitute for milk proteins—choose fortified products and monitor the nutritional value of the diet to avoid nutritional deficiencies;	for infants and young children
as a source of calcium and some vitamins, especially vitamin D and B_12_ (fortified beverages) for people on vegan diets or those who eliminate animal products due to religious, cultural, globalization-related, and ethical beliefs and considerations	in case of celiac disease, gluten allergy or intolerance only gluten-free fortified plant-based beverages are appropriate	
in case of lactose malabsorption or intolerance can be a substitute for milk and milk beverages (fortified beverages)		

**Table 9:** Recommended approaches regarding introduction of plant-based beverages for specific target groups.

Infants
main source of nutrition: breastfeeding or infant formula; plant-based beverages cannot be an alternative to breast milk or infant formula; in case of cow’s milk protein allergy, lactose-free soy formula (not plant-based soy drink) or rice protein hydrolysates can be considered, but are not first-choice options; for lactose intolerance, galactosemia, and congenital lactose deficiency, lactose-free soy formulas (not plant-based soy drinks) for infants can be used.
**Young Children**
preferred: whole cow’s milk, modified milk, or young child formula for children aged >1–3 years; routine use of plant-based beverages is not recommended. They cannot replace dairy products in a young child’s diet. In cases where plant-based beverages frequently or completely replace cow’s milk in the diet, the risk of vitamin or mineral deficiencies should be evaluated by a pediatrician or dietitian.
**Adults, People over 65 Years of Age, Adolescents**
adolescents and adults can consume plant-based beverages, but they should choose enriched drinks and pay attention to balancing their diets; calcium-fortified soy beverages, due to their comparable protein and calcium content to cow’s milk, may be an alternative to milk in the diets of the elderly avoiding/reducing milk consumption; because of reduced thirst in some elderly people, consumption of plant-based beverages can play a hydrating role; plant-based beverages, such as oat, almond, and rice drinks, have a lower protein content compared with milk and soy drinks, so their consumption should be supplemented with other sources of dietary protein to prevent sarcopenia and loss of muscle mass; among plant-based beverages, those fortified with calcium and vitamins, especially vitamin D and B_12_, are preferred.

**Table 10:** General information and advice regarding plant-based beverages for consumers.

a doctor and/or dietitian should be consulted before deciding to include plant-based beverages in the diet of healthy people from sensitive groups such as children, pregnant and breastfeeding women, the elderly, and people with medical conditions should consult a doctor and/or dietician before deciding to include plant-based beverages in the diet;
beverages fortified with calcium and vitamins, especially vitamin B_12_, B_2_, and D are preferable; the organic methods of processing raw materials and obtaining plant-based beverages limit the use of enrichment, according to EU regulations (Regulation (EU) 2021/1165; Regulation (EU) 2018/848; OJ C 278, 12.07.2021);
plant-based beverages without added sugars are preferable; sources of simple sugars, such as sucrose (beet and cane sugar), grape syrup, or agave syrup, should be avoided;
plant-based beverages should not be used in infant nutrition;
plant-based beverages should be shaken before consumption, as sediment containing calcium and starch may form at the bottom;
in the future, plant-based fermented beverages may be an alternative for consumers using plant-based beverages in their diet.

## Data Availability

No new data were created or analysed in this study. Data sharing is not applicable to this article.
